# The Metabolic Implications of Glucocorticoids in a High-Fat Diet Setting and the Counter-Effects of Exercise

**DOI:** 10.3390/metabo6040044

**Published:** 2016-12-05

**Authors:** Emily C. Dunford, Michael C. Riddell

**Affiliations:** School of Kinesiology and Health Science, Faculty of Health, Muscle Health Research Center and Physical Activity and Chronic Disease Unit, York University, 4700 Keele Street, Toronto, ON M3J 1P3, Canada; edunford53@hotmail.com

**Keywords:** glucocorticoids, high-fat diet, exercise, skeletal muscle, liver, adipose, metabolism

## Abstract

Glucocorticoids (GCs) are steroid hormones, naturally produced by activation of the hypothalamic-pituitary-adrenal (HPA) axis, that mediate the immune and metabolic systems. Synthetic GCs are used to treat a number of inflammatory conditions and diseases including lupus and rheumatoid arthritis. Generally, chronic or high dose GC administration is associated with side effects such as steroid-induced skeletal muscle loss, visceral adiposity, and diabetes development. Patients who are taking exogenous GCs could also be more susceptible to poor food choices, but the effect that increasing fat consumption in combination with elevated exogenous GCs has only recently been investigated. Overall, these studies show that the damaging metabolic effects initiated through exogenous GC treatment are significantly amplified when combined with a high fat diet (HFD). Rodent studies of a HFD and elevated GCs demonstrate more glucose intolerance, hyperinsulinemia, visceral adiposity, and skeletal muscle lipid deposition when compared to rodents subjected to either treatment on its own. Exercise has recently been shown to be a viable therapeutic option for GC-treated, high-fat fed rodents, with the potential mechanisms still being examined. Clinically, these mechanistic studies underscore the importance of a low fat diet and increased physical activity levels when individuals are given a course of GC treatment.

## 1. Introduction

Glucocorticoids (GCs) are a classification of steroid hormones, naturally produced by activation of the hypothalamic-pituitary-adrenal (HPA) axis, that are responsible for mediating both the immune system and energy metabolism. Over the past six decades, synthetic GCs have been established as an integral component of hematological cancer therapy [1] and are also used for the treatment of a variety of inflammatory conditions and diseases [2,3]. It is estimated that 1.0%–2.5% of the population of the United States aged ≥20 years (~2,513,259 persons) are prescribed oral GCs [4,5,6,7]. Unfortunately, chronic or high dose GC administration is associated with numerous side effects such as steroid-induced skeletal muscle loss (i.e., myopathy) [8], peripheral insulin resistance [9], visceral adiposity [10], and diabetes development [11]. GCs are often associated with excessive weight gain in humans and in animals [12], a feature that may be linked, at least in part, to an increased desire for energy dense “comfort” foods (e.g., high fat, simple sugars) [13,14]. A number of recent rodent studies have begun to investigate the relationship between poor dietary choices, such as a diet high in fat (HFD), in combination with chronic or high dose GC administration. It is possible that patients who are taking exogenous GCs could be more susceptible to poor food choices, but the effect of increasing fat consumption in combination with elevated exogenous GCs has only recently been investigated.

This paper will summarize the available information regarding the metabolic implications associated with the collective effects of elevations in GCs and a HFD (i.e., a diet specifically enriched with saturated fats). This information is critically important and clinically relevant as the number of patients taking exogenous GCs is rising and underscores the importance of a low fat diet when individuals are given a course of GC treatment.

## 2. Overview of GCs in Health and Disease 

Several cardiometabolic diseases (i.e., central obesity, type 2 diabetes, type 1 diabetes, metabolic syndrome, and pre-diabetes) are associated with elevations in GC concentrations [15]. The elevation in GCs, which may be the result of the disease itself or may be driving the disease progression, impacts a number of metabolic and behavior elements [16]. The glucocorticoid/glucocorticoid receptor (GC/GR) interaction plays a critical role in maintaining overall glucose homeostasis, in a number of tissues including the brain and periphery, as maintenance of systemic carbohydrate metabolism requires complex regulation between various peripheral organs in addition to the central nervous system. Subtle alterations in both cortisol secretion and/or tissue-specific reactivation of less active GCs (i.e., cortisone) to a more active form (i.e., cortisol), via the prereceptor enzyme 11β-hydroxysteroid dehydrogenase 1 (11β-HSD1), are a possible link between GC biology and the development of insulin resistance [17] and metabolic syndrome/type 2 diabetes mellitus (T2DM) [18,19,20].

**Figure 1:**
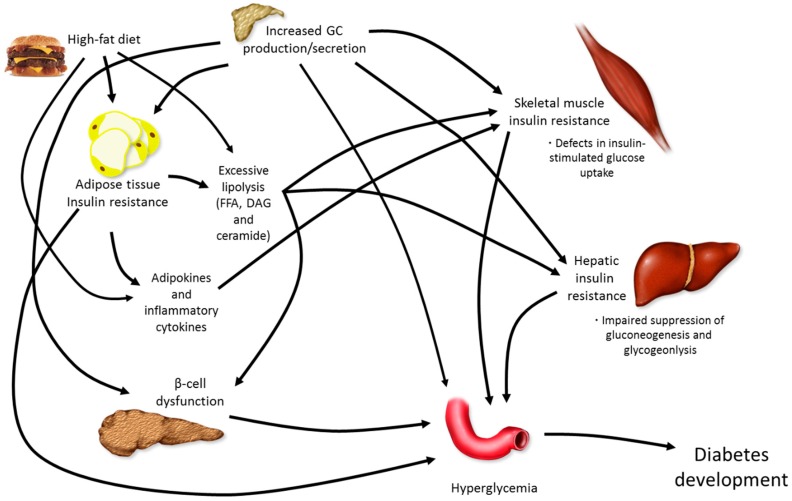
*Pathophysiology of diabetes development.* The development of overt diabetes occurs through a number of mechanisms, all of which work together to affect elevations in blood glucose, ultimately causing hyperglycemia. Glucocorticoids (GC), free fatty acids (FFA), diacylglycerol (DAG).

Due to their systemic catabolic actions, GCs increase hepatic glucose production [21], skeletal muscle protein degradation [22], and lipolysis [23]. As such, either endogenous overproduction (Cushing’s disease) or exogenous elevations in GCs via therapeutic treatment are associated with major metabolic disturbances, often culminating in T2DM development, particularly in a setting of increased caloric intake (Figure 1).

GCs act on the central nervous system to impact feeding behavior and physical activity patterns [14]. In rodents, elevations in GCs increase food intake in general, but tend to cause animals to consume sucrose and fats over high quality proteins or complex carbohydrates, perhaps because of an elevation in insulin levels [24]. Independently, chronically elevated GCs and the consumption of an energy dense diet containing saturated fat and/or simple carbohydrates cause dysregulated lipid metabolism within the skeletal muscle, liver, and adipose tissue of rodents and humans [25,26,27,28], promoting both increased visceral adipose mass accumulation and lipid deposition in various other non-adipose locations, such as the liver and skeletal muscle [10,23]. The increased ectopic fat deposition caused by an energy dense diet (in the face of relative inactivity) further propagates the detrimental aspects of the elevated catabolic action of GCs, through elevations in 11β-HSD1 activation and/or expression [29]. These detrimental changes appear to facilitate the production of fatty acid intermediates (ceramide and diacylglycerol (DAG)) in insulin sensitive tissues, such as skeletal muscle and liver, that inhibit specific proteins involved in insulin signaling [30]. Within the skeletal muscle, elevated GC exposure (or reactivation) reduces insulin-stimulated glucose uptake through inhibition of glucose transporter 4 receptor (GLUT4) translocation [31,32], accumulation of intramuscular lipids (IMCL), and increased fatty acyl-CoA production, subsequently increasing fatty acid intermediate concentrations. Within the liver, insulin resistance manifests as increased gluconeogenesis and glycogenolysis, thereby increasing endogenous glucose production. While both GCs and increased dietary fat consumption cause the proliferation of adipose tissue and adipose tissue hypertrophy, an altered pattern of adipokine secretion (i.e., increased leptin, decreased adiponectin, increased tumor necrosis factor α (TNFα), and elevated interleukin-6 (IL-6)) and increased lipolysis are also observed [33].

## 3. Metabolic Actions of GCs within the Skeletal Muscle, Liver, and Adipose Tissue

### 3.1. GCs Cause Dyslipidemia and Inhibit Insulin Signaling Proteins within the Skeletal Muscle

Insulin resistance, which is an impaired response of insulin-sensitive tissues to insulin signalling, is a characteristic feature of T2DM and plays a key role in the pathogenesis of the disease [34,35]. Systemic insulin sensitivity, under postprandial conditions, is mostly driven by skeletal muscle insulin sensitivity, although the liver also plays a role [36]. Hyperinsulinemia, even at physiologic levels, may actually stimulate a further worsening of insulin sensitivity in diabetes, thereby promoting a vicious cycle that places an unrelenting demand on pancreatic β-cell function [36].

Cushing’s disease patients are characterized by a redistribution of body fat from peripheral subcutaneous depots, to more central abdominal regions [37]. This over activity of the HPA axis, which is also seen with obesity [38], could be causally related to insulin resistance and diabetes development through ectopic lipid deposition (i.e., muscle, liver). GCs increase whole body lipolysis which leads to elevated levels of nonesterified fatty acids (NEFA) and triglycerides (TG) [39]. Elevations in NEFA concentrations increase the risk of accumulation of IMCL, fatty acyl CoA, DAG, and ceramide which inhibit select proteins along the insulin signalling pathway [30,40], ultimately suppressing glucose uptake, primarily through the inhibition of GLUT4 translocation to the cell membrane [41]. IMCL may activate various serine kinases, such as c-Jun amino-terminal kinase (JNK) and IkB kinase (IKK), which phosphorylate serine residues on insulin receptor substrate (IRS)-1, causing a decrease in insulin signaling [42]. IRS-1 is responsible for transmitting signals from both insulin and insulin like growth factor-1 (IGF-1) through the activation of the phosphatidylinositol (PI)-3 kinase (PI3K) and MAP kinase pathways. Elevated muscle ceramide levels, as a result of excessive ectopic lipid deposition from high fat feeding (HFF) or from high GC exposure, may be key mediators of insulin resistance and muscle atrophy [43], as these lipid metabolites are capable of directly inhibiting protein kinase B (Akt) phosphorylation [44].

GCs affect several integral proteins responsible for insulin-stimulated glucose uptake. Treatment of male rats with dexamethasone (Dex), a GR agonist, causes a reduction in insulin receptor (IR) phosphorylation within skeletal muscle and subsequent inhibition of Akt phosphorylation/activity after insulin administration [45]. This deleterious effect on muscle is likely direct, since muscle insulin signaling is also suppressed with GC treatment in cultured C2C12 myotubes [46]. Chronic elevations of GCs in rodents have also been found to induce both a decrease in tyrosine phosphorylation and total IRS-1 protein content [47,48]. GCs also reduce PI3K activity [49], as the GR seems to compete with IRS-1 for the association of PI3K subunits p110 and p85 [31,50]. As such, activation of the GR causes a reduction in IRS-1-associated PI3K activity and subsequent Akt phosphorylation at both serine and threonine sites [31,50]. The down-regulation in Akt activity has the potential to reduce GLUT4 translocation [41], impair glycogen synthesis, and limit skeletal muscle growth, via decreased mammalian target of rapamycin (mTOR) activity [51]. GC-induced atrophy has been shown to preferentially target fast, glycolytic muscle fibers (particularly type IIx and IIb) with almost no effect in type I fibers [52]. The reduction of skeletal mass by GCs occurs through both decreased protein synthesis and increased protein breakdown [53,54]. GC excess can also impair glycogen synthesis through suppressed glycogen synthase activity [31,32,41]. In line with much of this, GR mRNA levels in the skeletal muscle of diabetic patients correlate strongly with the degree of insulin resistance, with a normalisation of GR expression following pharmacological treatment that improves insulin sensitivity [55]. Interestingly, GC provision to aged rodents has a much more profound effect on skeletal muscle morphology and insulin sensitivity when compared to GC provision to younger rats [56]. As such, the detrimental effects of GCs may be augmented with aging, which could, at least in theory, contribute to age-related sarcopenia.

### 3.2. GCs Cause Hepatic Insulin Resistance and Dyslipidemia

The liver is a major organ responsible for regulating glucose and lipid homeostasis. In the fasted state, the liver’s glucose production through gluconeogenesis (GNG) and glycogenolysis (GGL) helps maintain euglycaemia. In the fed state, increased glucose uptake into the liver and the reduction in glucose production are critical components to post-prandial glucose control. With increased plasma glucose concentration, insulin is responsible for suppressing endogenous glucose production [57], while GCs and glucagon increase hepatic glucose output, which is the normal response in a fasting or catabolic state (e.g., prolonged exercise). In the presence of elevations of GCs, hepatic glucose production is elevated directly via activation of the enzymes responsible for glucose production (see below) and indirectly by promoting increased hepatic insulin resistance [58,59]. 

GC-induced hepatic insulin resistance results in impaired suppression of hepatic glucose production by insulin [60]. Generally, GCs exert their actions through the transcriptional regulation of a number of genes involved in hepatic carbohydrate metabolism, including glucose-6-phosphatase (G6Pase) and phosphoenolpyruvate carboxykinase (PEPCK), the rate-limiting enzymes involved in GNG [61,62]. Both of these enzymes contain a glucocorticoid response element (GRE) in their promoter regions which allows for GCs to directly modify their gene expression. As GCs are inherently catabolic, they are also capable of breaking down both protein and fat stores, ultimately increasing the availability of substrates for GNG. Moreover, similar to what is observed within the skeletal muscle, GC-induced elevation in lipid deposition in the viscera increases the supply of plasma NEFA and TG to the liver, which indirectly induces hepatic insulin resistance [63]. This accumulation of lipids within the liver, also known as hepatosteatosis, can lead to non-alcoholic fatty liver disease (NAFLD) [28], and possibly more severe liver diseases such as non-alcoholic steatohepatitis, fibrosis, cirrhosis, and cancer [64].

### 3.3. GCs Increase Adipose Tissue Proliferation and Lipolysis

It is clearly established that elevations in GCs, whether in Cushing’s disease patients or through exogenous corticosteroid therapy, cause excess adiposity [65]. This finding is somewhat surprising as GCs appear to exert catabolic actions in all other metabolically active tissues (i.e., skeletal muscle, liver, and bone) [66]. Within the adipose tissue itself, however, GCs have been shown to have a contradictory role in lipid metabolism, causing both increased lipolysis and increased visceral adiposity [23]. Generally, GCs increase lipolysis in mature adipocytes through enhanced transcription and expression of the lipase proteins adipose triglyceride lipase (ATGL) and hormone sensitive lipase (HSL) [67,68], while increased central adiposity occurs through upregulation of adipose cell proliferation in addition to increased food consumption [69]. Whether GCs alter changes in catecholamine-stimulated lipolysis is somewhat unclear [23].

As mentioned above, 11β-HSD1, which is predominately expressed within the liver, brain, skeletal muscle, and adipose tissue, amplifies the amount of active GCs in a given tissue, potentially increasing the exposure to increased GC activity [70]. Increased levels of 11β-HSD1 have been observed within the adipose depots of obese individuals [71] and further research has identified that both 11β-HSD1 [72] and GR [73] levels are much greater within the viscera when compared to the subcutaneous adipose depots. When compared to subcutaneous adipocytes, visceral adipocytes appear to have a higher affinity to GCs [74]. Increased 11β-HSD1 expression in adipose tissue in a transgenic rodent model resulted in elevated adipose tissue GC levels, enhanced food intake, and an accumulation of visceral adiposity, along with other features of metabolic syndrome [75]. This suggests that adipose tissue-specific increases in GC action, perhaps through the upregulation of 11β-HSD1 expression or activity, stimulates enhanced visceral fat accumulation and deteriorates whole body insulin sensitivity via direct actions within the adipose tissue. 

## 4. GCs and Food Intake

### Impact of GCs on Brain and Feeding Behaviour

The catabolic activity of GCs causes the mobilization of substrates from peripheral sources such as the skeletal muscle, liver, and adipose tissue. GCs are also capable of acting on the brain by modulating behaviour, autonomic, and sympathetic responses in addition to augmenting learning and memory [76]. The consumption of foods containing high fat and high sugar substances (often called “comfort foods”) has been found to be proportional to circulating GCs, and corticosterone is a hormone known to stimulate food-associated drives or motivations [14,77]. GCs also stimulate insulin secretion, and along with the resultant hyperinsulinemia, this interaction of GCs with hyperinsulinemia, modulates food choice to favour sucrose and lard in rodents [5,16].

Two elegant rodent studies examined the effect of prior metabolic stress (restraint or cold stress) on the preference for “comfort foods” and the subsequent response of the HPA axis. Both studies found that “comfort” foods were preferred to standard chow, and the provision of these “comfort” foods reduced the degree of the stressor-induced sympathetic responses and lowered the basal corticotropin-releasing factor/hormone (CRF) levels within the hypothalamus [78,79]. Cushing’s disease patients have been found to choose high-fat foods, when compared to individuals with normal GCs levels [80]. Moreover, elevations in catecholamines, which are also associated with heightened HPA axis activity, have been shown to increase feeding activities in rodents [81]. GCs indirectly increase feeding behaviour through the modification of neuropeptide Y (NPY) [82], the key orexigenic neurotransmitter linked to food consumption and adipose tissue deposition [83].

## 5. Combining Elevations in GCs with High-Fat Feeding

### 5.1. Dexamethasone or Corticosterone Exogenous Treatment and High-Fat Feeding

With the number of people currently taking synthetic GCs on the rise [84], it is important to fully understand the association between elevations in GC concentrations and increased dietary fat consumption. Specifically, the type of fat is critically important, as diets high in saturated fatty acids are a more potent diabetogenic stimulus than mono- and polyunsaturated fatty acids [85]. Surprisingly, very limited research has been conducted examining the potential synergistic actions of the two metabolic insults. Moreover, the effects of high quality protein intake on attenuating GC-induced muscle loss and insulin sensitivity are unknown.

**Figure 2:**
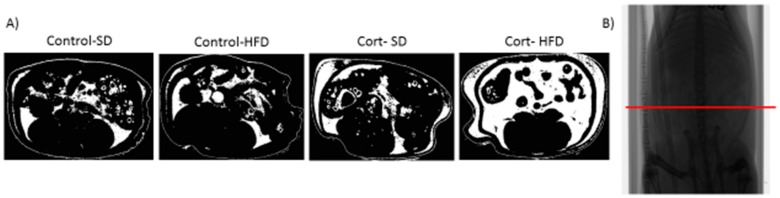
*Micro-computed tomography shows that corticosterone and a high-fat diet caused increased abdominal fat.* (**A**) A representation of abdominal fat from a representative rat from each group is shown; (**B**) A whole rat radiography with the ROI (region of interest) used. Standard diet (SD), high-fat diet (HFD), corticosterone (Cort).

In one key study, Gounarides et al. [86] examined the effect of three intraperitoneal injections of Dex over 5 days, in a diet-induced obesity (DIO) murine model of insulin resistance (2 months of HFD; 45% of the total calories from lard; D12451, Research Diets). The authors found that compared to controls, the DIO and Dex-treated mice had impaired glucose tolerance, increased food consumption, body weight, visceral adiposity, and skeletal muscle lipid deposition, in addition to a shift in lipid profile towards more saturated fats. Additional work examined the effect of HFD (enriched with 17.5% soybean oil), adrenalectomy, and Dex treatment on the expression of adiponectin and its receptors in another complementary study [87]. GC exposure in wild-type mice is known to cause inhibition of adiponectin receptor gene expression within skeletal muscle [88], possibly affecting positive downstream consequences of adiponectin signaling in skeletal muscle, such as fatty acid oxidation and the prevention of IMCL accumulation. With the addition of a HFD following adrenalectomy and subsequent Dex treatment, both circulating adiponectin and adiponectin receptor concentrations were decreased within the skeletal muscle, suggesting one more potential deleterious metabolic effect when the two metabolic insults are combined [87]. A more recent rodent model utilizing corticosterone pellet implants, in combination with HFD (60% of the total calories from lard, D12492, Research Diets; abbreviated to Cort-HFD) has been developed to induce a rapid diabetic phenotype in young Sprague-Dawley rats [89]. The Cort-HFD rats become severely hyperglycemic, hyperinsulinemic, and insulin resistant through the development of hepatic steatosis [90], impaired β-cell function [91], and increased lipid deposition in the viscera, liver, and skeletal muscle [89,92] (Figure 2). In another model, broiler chicks received either a HFD (enriched with 13.5% soybean oil) or low-fat diet (0% soybean oil; LFD) for 6 days and were then given an intracerebroventricular injection of either Dex or saline. The Dex injection, in combination with the 6 days of HFD, caused significant decreases in hypothalamic gene expression, which were not observed in any other treatment conditions [93]. What is interesting about all of these models is that with corticosterone/dexamethasone or a HFD alone, the animals do not develop the same metabolic impairments, but when the two treatment conditions are combined, the detrimental actions are dramatically amplified. These animal models seem to mimic many of the symptoms observed in patients with T2DM, and provides evidence that elevations in GCs induce diabetes development, especially if the susceptible individuals do not consume a normal low fat diet.

Generally, the above HFD/GC overexposure models demonstrate a consistent and rapid increase in both visceral adiposity and ectopic lipid deposition (liver, muscle). It is possible that since elevations in IMCL are known to occur within 2–3 days of HFD [94], and as both GCs and a HFD cause significant IMCL accumulation, it could be proposed that the rapidity of the metabolic dysfunction, within the majority of these co-treatment models, could be attributed to the initial accumulation in IMCL. When combined with augmented tissue-specific GC activity, as 11β-HSD1 content is increased in the adipose tissue with HFD, the co-treatment could be both increasing visceral adiposity and active GC exposure within the adipose tissue, propagating the detrimental effects of the elevated GCs. Further work examining the mechanisms which contribute to the augmentation of the deleterious metabolic effects caused by the combination of elevations in GCs, whether exogenously or endogenously, and HFD is necessitated. 

### 5.2. Chronic Stress and High-Fat Feeding

Another methodology utilized to induce HPA activation is the chronic stress model, such as chronic social defeat stress (CSDS), restraint stress, or cold stress. Generally, when this form of GC elevation is combined with a HFD, the results are surprisingly contradictory to what is observed when Dex or corticosterone is injected or implanted exogenously. In mice exposed to both DIO and CSDS, the chronic stress actually improved the increased adiposity, body weight, and glucose intolerance observed after DIO [95]. Similarly, Wistar rats provided with a HFD and immobilized for 1 h, 5 days/week for 15 weeks, were protected from DIO, as these animals consumed less, and when compared to the non-stressed, DIO rats, had normal glucose tolerances [96]. Clinically, this is very relevant, and needs to be further explored as it suggests that those individuals taking exogenous GC therapy are at much higher risk of further metabolic complications if consuming a diet high in fat. 

## 6. Role of Exercise in Offsetting the Metabolic Implications of Both Elevated GCs and High-Fat Feeding

Regular physical activity (i.e., exercise) is widely used to control and/or treat several cardiometabolic diseases because of its compensatory and regulatory actions on a variety of organs including muscle, bone, liver, and adipose tissue. Interestingly, exercise is itself a common form of metabolic stress which stimulates the HPA axis [97] causing increased circulating GCs to mobilize fuel sources and subdue the immune system during exercise-induced muscle damage. While elevations in circulating GC levels induce insulin resistance, regular exercise enhances insulin sensitivity and causes reductions in both GR and IIβ-HSD1 expression within the skeletal muscle, liver, and adipose tissue [98], ultimately reducing tissue-specific GC exposure. Regular treadmill running before and during two weeks of Dex treatment improved glycogen loss [99,100], hyperinsulinemia, and muscular atrophy [100] in some muscles, as well as increasing physical capacity. When analyzed in rheumatoid arthritis patients, 24 months of dynamic strength training improved both muscle strength and physical function, without negatively impacting disease activity [101]. While others have found, in rodents, both chronic treadmill running and resistance training downregulate muscle RING-finger protein-1 (MuRF1) and atrogin-1 in atrophy induced through elevations in circulating GCs, in addition to improving glucose homeostasis [102,103,104,105]. The best form of exercise to limit GC-induced metabolic dysregulation is unknown, but typically both resistance and aerobic forms of exercise are thought to have independent and additive beneficial effects, at least in humans [106,107]. High-intensity resistance training has recently been demonstrated to attenuate dexamethasone-induced muscle atrophy in rats via up-regulation of mTOR and p70S6K and a reduction in MuFR-1 protein expression [108].

Voluntary exercise, generally considered to be aerobic, is well known to improve skeletal muscle insulin-stimulated glucose uptake [109] and insulin resistance [99], induced by GC exposure, through attenuation of reductions in specific insulin signaling proteins, including Akt and IRS-1 activity and expression. Exercise also lowers ceramide content, while at the same time improving insulin sensitivity, possibly by directing lipids into the triglyceride pool [110,111,112]. However, exercise training is not typically able to improve the extreme weight loss seen with dexamethasone or corticosterone treatment [113] or some of the reductions observed in angiogenesis, specifically decreased vascular endothelial growth factor (VEGF) expression [100]. 

**Figure 3:**
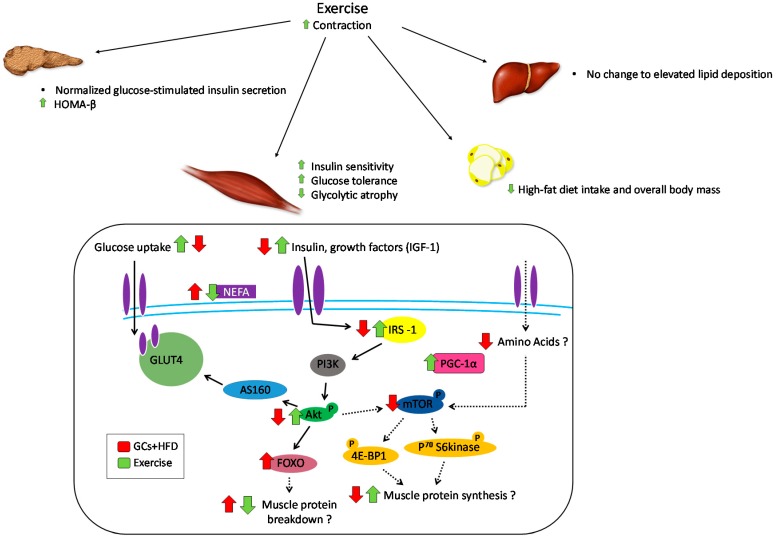
*Counter-effects of exercise on metabolic impairments caused by elevated glucocorticoids and high-fat diet.* Physical activity (treadmill running or voluntary wheel running) is capable of improving some of the metabolic effects caused by elevated glucocorticoids (GCs) and a high-fat diet (HFD), specifically within the pancreas, skeletal muscle, adipose tissue, and liver. The skeletal muscle is currently the most researched, and the majority of the benefits of exercise revolve around improvements in insulin signaling. Elevations in GCs combined with HFD cause significant metabolic effects, and further examination of exercise on the pancreas, adipose tissue, and liver is required.

Remarkably, very little is known in regard to the effect of regular exercise in rodents or humans consuming a HFD while undergoing exogenous GC treatment. To date, only three studies have investigated the therapeutic potential of exercise, predominantly aerobic exercise, in GC-treated, high-fat fed rodents. Liu et al. [114] established a mouse model which expressed a depression-like phenotype and an insulin-resistant state through the chronic treatment of corticosterone and HFD (Cort-HFD). Following 4 weeks of treadmill running, the Cort-HFD mice had improved blood glucose concentrations, decreased depression-like symptoms and overall improvements in mobility [114]. Similar results were observed in Cort-HFD treated rats following 4 weeks of voluntary wheel running. The Cort-HFD rats showed improved glucose tolerance, insulin secretion, hyperglycemia, hyperinsulinemia, and homeostatic model assessment of insulin resistance (HOMA-IR) values [92]. There were also specific skeletal muscle improvements, with increased glycolytic muscle mass, tibialis anterior cross-sectional area, oxidative capacity, and capillarization (Figure 3). An interesting aspect to this study was that these results were exercise volume specific, as less running correlated with a reduced amount of metabolic improvements, highlighting the fact that exercise-induced adaptations depend on the duration, intensity, and overall volume of the exercise intervention. Another study investigated the effect of Cort provision during voluntary wheel running on HFD preference [115]. Sprague-Dawley rats that were provided Cort in their drinking water had reduced HFD intake and body weight during wheel running, but interestingly no change in HFD preference. These results suggest that voluntary exercise is able to decrease the preference and overall intake of HFD in rats, and that this effect is possibly amplified when exogenous Cort is administered. Clearly, further work is required to fully elucidate all of the possible benefits associated with voluntary exercise (both resistance- and endurance-type exercise) and alterations in macronutrient content of diet (e.g., high-quality protein diet vs. high fat diet) in both humans and rodents undergoing exogenous GC therapy.

## 7. Conclusions

Due to their pleiotropic consequences, GCs affect almost every tissue in the body, which increases their possible therapeutic benefits, but also intensifies likely side effects. Synthetic GCs are the predominant form of treatment for numerous inflammatory and immunological disorders, which makes the understanding the effect of diet and nutrition on their action a major clinical concern. Significant relationships exist between exogenous elevations in GC concentrations, peripheral insulin resistance, and diabetes development; an association which becomes magnified with the addition of a HFD. Fortunately, exercise appears to be a viable therapeutic option to offset some of the more severe metabolic side effects by improving overall glucose tolerance, skeletal muscle insulin signaling, insulin secretion, and body composition. Further research is required to fully understand the metabolic consequences of combining elevations in GCs and increased dietary fat in addition to the effect of exercise as a possible countermeasure.
